# Characterization and Evaluation of the Pathogenicity of a Novel Alpha Family Chaperone‐Usher Pilus Present on Porcine Enterotoxigenic *Escherichia coli*

**DOI:** 10.1155/tbed/9901219

**Published:** 2026-08-25

**Authors:** Yi-Fang Lai, Yen-Chen Chang, Wei-Ting Lee, Shang-Te Danny Hsu, Chao-Nan Lin, Tzu-Ming Huang, Chien-Yu Chen, Dung-Chi Wu, Hao-Sen Chiang, Hui-Wen Chang

**Affiliations:** ^1^ School of Veterinary Medicine, National Taiwan University, Taipei 10617, Taiwan, ntu.edu.tw; ^2^ Graduate Institute of Molecular and Comparative Pathobiology, National Taiwan University, Taipei 10617, Taiwan, ntu.edu.tw; ^3^ Institute of Biological Chemistry, Academia Sinica, Taipei 11529, Taiwan, sinica.edu.tw; ^4^ Institute of Biochemical Sciences, National Taiwan University, Taipei 10617, Taiwan, ntu.edu.tw; ^5^ Department of Veterinary Medicine, College of Veterinary Medicine, National Pingtung University of Science and Technology, Pingtung 91201, Taiwan, npust.edu.tw; ^6^ Biology Division, Veterinary Research Institute, Ministry of Agriculture, Tamsui, New Taipei City, Taiwan, moa.gov.cn; ^7^ Department of Biomechatronics Engineering, National Taiwan University, No. 1, Sec. 4, Roosevelt Road, Taipei 10617, Taiwan, ntu.edu.tw; ^8^ Genome and Systems Biology Degree Program, National Taiwan University and Academia Sinica, Taipei 10617, Taiwan; ^9^ Department of Life Science, National Taiwan University, Taipei, Taiwan, ntu.edu.tw

**Keywords:** antimicrobial resistance, chaperone-usher, enterotoxigenic *Escherichia coli*, intestinal tropism, neonatal piglets, whole-genome sequencing

## Abstract

Enterotoxigenic *Escherichia coli* (ETEC) is a leading cause of diarrhea in piglets, imposing significant economic burdens on swine production worldwide. Despite its impact, detailed characterization of intestinal colonization and comprehensive genomic profiling of virulent neonatal porcine ETEC strains remain limited. In this study, we isolated a β‐hemolytic ETEC strain, PT4357, from a diarrheic neonatal piglet and conducted whole‐genome sequencing (WGS) coupled with experimental infection in neonatal piglets to define its pathogenicity. The PT4357 belonged to serotype O8:H7 and sequence type ST2521 and harbored multiple virulence and antimicrobial resistance (AMR) genes, including the plasmid‐borne colistin resistance gene *mcr-1*. Interestingly, a novel chaperone‐usher (CU) assembly pathway pilus has a homolog of the α family usher gene that was identified in the chromosome of PT4357. Compared with the CS1 pilus on ETEC, structural superposition of the CU pathway pilus of PT4357, namely, CS33, showed strong conservation in the major subunit and chaperone and was relatively divergent in the N‐terminal of usher and minor subunits while retaining essential core folds. Oral inoculation confirmed villi attachment of the ETEC PT4357, causing watery diarrhea in neonatal piglets without mucosal damage. Furthermore, the adherence of the ETEC PT4357 on porcine IPEC cells but not human Caco‐2 cells suggests limited human tropism. Our results indicate that Taiwan porcine ETECs carried AMR genes and alternative CU pathway pilus. The role of the CS33 pilus on the pathogenicity or virulence of the pathogen requires further investigation and surveillance.

## 1. Background

Swine colibacillosis refers to infections caused by *Escherichia coli* (*E. coli*) in pigs and encompasses a range of clinical syndromes, including neonatal and postweaning diarrhea (PWD), edema disease (ED), septicemia, polyserositis, coliform mastitis, and urinary tract infections [1]. It is widely recognized as a major bacterial disease affecting the global swine industry, resulting in substantial economic losses attributable to morbidity and mortality, impaired growth performance, and increased expenditures for disease prevention, therapeutic interventions, and herd management, such as vaccination programs and feed supplementation strategies [2].

Among the diverse *E. coli* strains, such as enterotoxigenic *E. coli* (ETEC), shiga toxin–producing *E. coli* (STEC), and enteropathogenic *E. coli* (EPEC), classified according to their virulence factors, ETEC is the principal etiological agent of intestinal disease in pigs, causing neonatal and PWD [3]. Strains of ETEC generally present specific fimbrial adhesins (F), which are long proteinaceous appendages extending from the bacterial surface, such as F4 (K88) and F18, that mediate adherence to enterocytes on the intestinal mucosa. Following colonization, bacterial proliferation occurs, accompanied by the production of enterotoxins, including heat‐labile toxin (LT) and/or heat‐stable toxin (ST). The LT activates adenylate cyclase, leading to increased intracellular cyclic adenosine monophosphate (cAMP), whereas ST stimulates cyclic guanosine monophosphate (cGMP) signaling in intestinal epithelial cells, ultimately resulting in excessive fluid secretion into the intestinal lumen [4]. Clinically, ETEC‐infected piglets typically present with watery diarrhea, dehydration, severe weight loss, and, in severe cases, death [1]. The expression of F4 and F18 receptors on the brush border of the small intestine is age‐dependent, which largely explains the predominance of F4‐positive ETEC in neonatal and early postweaning pigs and F18‐positive ETEC in later postweaning stages [5].

In recent years, a variety of in vivo ETEC challenge models have been developed and widely applied to evaluate the efficacy of therapeutic and preventive interventions. Oral inoculation of neonatal or weaned piglets with ~10^9^ CFU of pathogenic *E. coli* reproducibly induces diarrhea within hours to days postchallenge, with earlier onset and increased disease severity observed following infection with STa^+^ strains and in immunologically naïve animals [6–8]. However, despite the consistent induction of clinical disease, the associated pathological features and intestinal colonization patterns of neonatal ETEC infection have not been systematically characterized in vivo.

In the present study, a pathogenic ETEC strain, PT4357, was isolated from neonatal piglets with diarrhea. The pathogenicity and intralesional distribution of PT4357 were subsequently investigated in 4‐day‐old piglets using an experimental infection model. The virulence gene profile of the isolate was determined using multiplex polymerase chain reaction (PCR), and its complete genome was characterized by next‐generation sequencing (NGS). Interestingly, a novel chaperone‐usher (CU) pathway pilus, namely, CS33, with an usher protein homolog of the α family was identified and characterized using phylogenetic analysis. The deduced major, minor, chaperon, and usher protein sequences of the CS33 pilus were aligned with the CS1 CU pilus and homology‐modeled by AlphaFold3. The attachment of ETEC PT4357 in porcine IPEC cells was compared with human Caco‐2 cells. By delineating the clinical outcomes and pathological manifestations and comparing the cell attachment tropisms of the CS33 pili–bearing ETEC PT4357 in human and porcine enteric cell lines, this study aims to provide insights into the novel CU pathway pili–bearing porcine ETEC.

## 2. Material and Methods

### 2.1. Bacterial Strain and Detection of Virulence Genes

The pathogenic *E. coli* strain PT4357 used in this study was isolated from a neonatal pig exhibiting watery diarrhea at the Animal Disease Diagnostic Center, National Pingtung University of Science and Technology, Taiwan. Virulence genes encoding F4, F5, F6, F18, F41, STa, STb, LT, STx2e, and Intimin were detected using multiplex and conventional PCR according to the primers and protocols as previously described [9, 10]. Briefly, the multiplex PCR reaction mixture contained 25 µL of AmaR OnePCR (GeneDireX, Taipei, Taiwan), 1 µL of each forward and reverse primer (10 mM), a single bacterial colony grown on tryptic soy agar with 5% sheep blood (Dr. Plate Biotech, Taipei, Taiwan), and nuclease‐free distilled water, bringing the final volume to 50 µL. PCR amplification was performed using a T100 Thermal Cycler (Bio‐Rad Laboratories, California, USA) with the following program: initial denaturation at 94°C for 5 min, 35 cycles of 94°C for 40 s, 55°C for 1 min, and 72°C for 2 min, followed by a final extension at 72°C for 5 min. Amplicons were separated by 2% agarose gel electrophoresis, stained with ethidium bromide, and visualized using a UV transilluminator and the Gel Doc XR Imaging System (Bio‐Rad, Hercules, CA, USA).

For bacterial inoculum preparation, PT4357 was cultured in sterile LB broth without antibiotics at 37°C for 16–18 h with shaking at 200 rpm. The bacterial concentration was estimated by measuring optical density at 600 nm (OD600) using a WPA Biowave II spectrophotometer (Biochrom, Cambridge, UK), with 10‐fold dilutions. The bacterial stock for inoculation was adjusted to a final concentration of 5 × 10^8^ CFU/mL.

### 2.2. Antimicrobial Susceptibility Testing

The phenotypic antimicrobial susceptibility of the ETEC PT4357 strain was determined using the Kirby–Bauer disk diffusion method, as previously described [11]. Bacterial colonies were suspended in 3 mL of sterile saline and adjusted to 0.5 McFarland turbidity. The suspension was uniformly inoculated onto Mueller–Hinton agar plates (TPM Ready‐to‐Use Media, Taiwan). Each isolate was tested against multiple antimicrobial classes using commercial disks (BD, USA; Thermo Fisher Scientific, USA): penicillins (ampicillin 10 μg), β‐lactam combinations (amoxicillin–clavulanate 20/10 μg), cephalosporins (cefazolin 30 μg, cefoxitin 30 μg, and cephalexin 30 μg), carbapenems (meropenem 10 μg and imipenem 10 μg), quinolones (ciprofloxacin 5 μg), aminoglycosides (gentamicin 10 μg and amikacin 30 μg), sulfonamides (sulfamethoxazole/trimethoprim 23.75/1.25 μg), and phenicols (chloramphenicol 30 μg). The minimum inhibitory concentrations (MICs) of colistin, enrofloxacin, kanamycin, tetracycline, doxycycline, and florfenicol (Sigma–Aldrich, St. Louis, MO, USA) against the tested isolate were determined using the broth microdilution method in accordance with the European Committee on Antimicrobial Susceptibility Testing (EUCAST) reference methodology. Colistin sulfate was prepared in cation‐adjusted Mueller–Hinton broth (CAMHB) and serially diluted two‐fold in 96‐well microtiter plates to obtain final concentrations ranging from 0.125 to 256 μg/mL. Bacterial suspensions were adjusted to a turbidity equivalent to a 0.5 McFarland standard and further diluted in CAMHB to achieve a final inoculum concentration of ~5 × 10^5^ CFU/mL in each well. The microplates were incubated at 37°C for 18–20 h under aerobic conditions. The MIC was defined as the lowest concentration of colistin required to completely inhibit visible bacterial growth. All MIC experiments were performed in triplicate. MICs and zone diameters were interpreted according to the epidemiological cut‐off values (ECOFFs) established by the EUCAST Subcommittee on Veterinary Antimicrobial Susceptibility Testing (VetCAST). ECOFFs were obtained from the EUCAST MIC and zone diameter distribution database (https://www.eucast.org/vetcast/mic-distributions-and-ecoffs/). *E. coli* ATCC 25922 was used as a quality control strain. Multidrug‐resistance was defined as resistance to ≥1 agent in three or more antimicrobial classes [12].

### 2.3. Genomic DNA Extraction, Library Preparation, and Whole‐Genome Sequencing (WGS)

A single colony of PT4357 grown overnight on blood agar was suspended in sterile phosphate‐buffered saline (PBS), and genomic DNA was extracted using the DNeasy UltraClean Microbial Kit (Qiagen, Germany) following the manufacturer’s instructions. DNA concentration and purity were assessed using Cytation 7 (BioTek Instruments, Agilent Technologies, California, USA). Genomic DNA underwent DNA repair and end‐preparation using the NEBNext FFPE DNA Repair Mix and NEBNext Ultra II End Repair/dA‐tailing Module (New England Biolabs, Ipswich, MA, USA) following the manufacturer’s protocol.

### 2.4. Genome Assembly, Circularization, Polishing, and Annotation

WGS was performed on a MinION Mk1B (Oxford Nanopore Technologies, UK) using the Native Barcoding Kit (SQK‐NBD114.24) and MinION Flow Cell R10.4.1 (FLO‐MIN114). Base calling was performed with Dorado (v0.7.0; Oxford Nanopore Technologies) using the super‐accuracy (sup) model. De novo assembly and circularization were performed using Autocycler v0.5.2 [13]. The pipeline incorporated multiple long‐read assemblers, including Flye v2.9.6‐b1802, Raven v1.8.3, Miniasm v0.3‐r179, MetaMDBG v1.2, NECAT v0.0.1_update20200803, NextDenovo v2.5.2, Plassembler v1.8.0, and Canu v2.3. Assembly polishing was conducted using Racon v1.5.0. Read alignment was performed using Minimap2 v2.28‐r1209, and genome distance estimation was conducted using Mash v2.3. Assembly graphs were inspected using Bandage to confirm circularization. Contigs from the previous assembly were annotated using the NCBI Prokaryotic Genome Annotation Pipeline (PGAP) with default parameters.

### 2.5. Bioinformatic Analysis

The mean genome‐wide coverage depth was ~150×. Virulence and antimicrobial resistance (AMR) genes, serotype, plasmid replicons, fimbrial types, mobile elements, and pathogenicity potential were identified using VirulenceFinder 2.0 (Center for Genomic Epidemiology, DTU) with a minimum nucleotide identity threshold of 90% and a minimum coverage threshold of 60%; ResFinder 4.7.2 with thresholds of 80% nucleotide identity and 60% minimum coverage; KmerFinder, SerotypeFinder, multilocus sequence typing (MLST), PlasmidFinder, FimTyper, MobileElementFinder, KmerResistance, and PathogenFinder 2.1 with thresholds of 95% nucleotide identity and 60% minimum coverage on the Center for Genomic Epidemiology platform (https://cge.food.dtu.dk/). AMR genes were further confirmed against the Comprehensive Antibiotic Resistance Database (CARD).

### 2.6. Phylogenetic Analysis of the Usher Protein of the CS33 CU Pathway Pili

**Figure 1:**
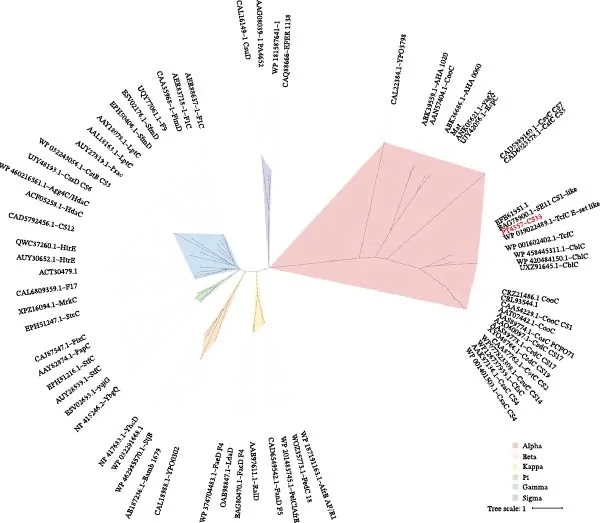
Phylogenetic analysis of the deduced usher protein sequence of CS33 pili of ETEC PT4357 with 72 usher sequences of chaperone‐usher pathway pili in six clades. Sequences were aligned using ClustalW. Phylogenetic analysis was performed with MEGA12 software. The Neighbor‐Joining method and 1000‐replicate bootstrap test were used to generate a phylogenetic tree, and the unrooted phylogram was displayed in iTOL. Fimbrial gene clusters were grouped according to the Nuccio clade system (α, β, γ, π, κ, and σ).

To further investigate the CS33 assembly pathway pili identified on the chromosome in our ETEC PT4357 strain, the amino acid sequence of the CS33 pilus usher protein of PT4357 was first aligned with a total of 72 reference strains belonging to six clades of CU assembly pathway sequences based on the Nuccio scheme [14] (Figure 1) using ClustalW [15]. Phylogenetic analysis was performed with MEGA12 software [16]. The Neighbor‐Joining method and 1000‐replicate bootstrap test were used to generate a phylogenetic tree, and the unrooted phylogram was displayed in iTOL [17].

### 2.7. Alignment and Homology Modeling by AlphaFold3

The nucleotide and corresponding amino acid sequences of the usher, chaperon, major, and minor proteins of CS33 pili of PT4357 were each aligned with respect to the CooC, CooB, CooA, and CooD of human ETEC ESEI_164 CS1 (GenBank Accession Number LN870269.1) using ClustalW. The sequences of usher, chaperon, major, and minor proteins of CS33 pili of porcine ETEC PT4357 and those in the human ETEC ESEI_164 CS1 were used as input for modeling by AlphaFold3 [18] (https://alphafoldserver.com/) using default settings. The output models were visualized and rendered by PyMOL (ver. 3.1.3.1; The PyMOL Molecular Graphics System, Version 3.0 Schrödinger, LLC.). The definitions of the individual domain boundaries followed the previous literature [19, 20]. For the rendering, the first 20 residues of the ternary complexes of our CS33 of PT4357 and CS1 were omitted as they likely correspond to the signal peptides that will be removed upon biosynthesis. Structure‐based multiple sequence alignment and conservation analysis were achieved by using ENDscript2.0 [21] using default settings. The AlphaFold model of the apo forms of CS33 usher served as the input to generate the sequence conservation scores, which were incorporated as B‐factors in the resulting model and visualized using PyMOL.

### 2.8. Detection of the Major Subunit Gene of CS33 Pili of PT4357 in Taiwan Porcine *E. coli* Field Strains

To investigate the prevalence of the novel CS33 CU pathway pili in porcine *E. coli* in the field, a total of 14 *E. coli* strains isolated from diarrheal or systemically infected pigs were submitted to the Yunlin Animal Disease Diagnosis Center, comprising 12 strains carrying ETEC, STEC, or EPEC virulence genes and two strains isolated from brain and lungs devoid of these virulence genes (Table 1). These *E. coli* strains were cultured on tryptic soy agar supplemented with 5% (v/v) defibrinated sheep blood at 37°C for 12–16 h. Presumptive *E. coli* colonies were confirmed by 16S rRNA sequencing. Genomic DNA was extracted using the DNeasy Blood and Tissue Kit (Qiagen, Germany) according to the manufacturer’s instructions. Virulence‐associated genes, including fimbrial adhesins and enterotoxins, were screened by multiplex PCR following previously described protocols with minor modifications. For the detection of the CS1‐like major subunit gene, primers were designed based on the WGS of the PT4357 strain using Geneious Prime. Primer sequences were as follows: forward 5′‐ATGAAAAAGGTATTTGCAAAATCTCTTC‐3′ (positions 2144933–2144960) and reverse 5′‐TTAGATATCCTGAGGACAGATAAATGC‐3′ (positions 2145408–2145433), yielding a 501 bp amplicon. PCR was performed under the following cycling conditions: initial denaturation at 95°C for 5 min; 35 cycles of 95°C for 30 s, 52°C for 30 s, and 72°C for 45 s; and a final extension at 72°C for 5 min. Amplified products were visualized on a 2% agarose gel and subsequently confirmed by Sanger sequencing.

**Table 1:** List of virulence‐associated genes of the ETEC PT4357 strain.

Functional category	Gene(s)	Predicted function	Locations
Adhesion, fimbrial attachment, and colonization‐associated factors	*faeB, faeC, faeE, faeF, faeG, faeH, faeI*	F4 fimbrial	Plasmid
	*fimA, fimH*	Type 1 fimbriae‐mediated adhesion	Chromosome
	*yehA, yehB, yehC, yehD*	Yeh fimbrial	Chromosome
	*papA_F19*	Major pilin subunit F19	Chromosome
	*lpfA*	Long polar fimbriae	Chromosome
	*tia*	Intestinal epithelial adhesion and invasion	Chromosome
	*fdeC*	Intimin‐like adhesin FdeC	Chromosome
	*yghJ*	Secreted metalloprotease (SslE/YghJ homolog)	Chromosome
Enterotoxins	*eltIAB-4*	Heat‐labile enterotoxin LTIh‐4	Plasmid
	*estb-STb1*	Heat‐stable enterotoxin STb1	Plasmid
	*astA*	Heat‐stable enterotoxin EAST‐1	Plasmid
Biofilm formation	*csgA*	Curli major subunit	Chromosome
Immune evasion and serum resistance	*iss*	Increased serum survival	Chromosome
	*traT*	Outer membrane protein complement resistance	Plasmid
Hemolysins	*hlyA*	Hemolysin A	Plasmid
	*hlyE*	Hemolysin E	Chromosome
Host adaptation and colonization‐associated functions	*shiA*	Homolog of the *Shigella flexneri* SHI‐2 pathogenicity island gene shiA	Chromosome
	*gad*	Glutamate decarboxylase	Chromosome
	*anr*	AraC negative regulator	Plasmid
Metal resistance	*terC*	Tellurium ion resistance protein	Chromosome
Conjugation‐associated functions	*traJ*	Positive regulator of conjugal transfer operon	Plasmid

### 2.9. Adhesion Assay

The human Caco‐2 (ATCC Number HTB‐37) and porcine IPEC cell lines were cultured in a monolayer with Dulbecco’s modified Eagle’s minimal (DMEM) essential medium (Thermo Fisher Scientific Inc., Waltham, MA, USA) supplemented with penicillin/streptomycin sulfate (Thermo Fisher Scientific Inc.) and 20% and 10% FBS, respectively (Millipore, Burlington, MA, USA). The Caco‐2 and IPEC cells were seeded in 24‐well tissue culture plates and grown to 90% confluence for subsequent assays. All culture incubations were performed in a humidified 37°C, 5% CO_2_ incubator (Thermo Fisher Scientific Inc.). Porcine ETEC strain PT4357 and the porcine ETEC NTU25‐033 (absence of CS33 major subunit) were grown on CFA agar overnight and resuspended in LB broth. The Caco‐2 cells and IPEC cells were inoculated with these two strains of bacteria at a multiplicity of infection of 10. Bacteria and Caco‐2/IPEC cells were coincubated in DMEM for 2 h at 37°C, washed three times with PBS, and then treated with 0.1% (vol/vol) Triton X‐100. The suspensions were serially diluted and plated on LB agar plates overnight. After incubation, the CFU of each plate was calculated. The assay was performed in triplicate. Statistical analyses were performed using two‐way ANOVA followed by Tukey’s multiple comparisons test in GraphPad Prism (version 11.0.2; GraphPad Software, San Diego, CA, USA).

### 2.10. Animal Experimental Design

Six 4‐day‐old neonatal piglets (Duroc × Landrace‐Large White) from sows without *E. coli* vaccination were used. Piglets were clinically healthy and tested negative by PCR for porcine epidemic diarrhea virus, porcine deltacoronavirus, rotavirus, transmissible gastroenteritis virus, and the virulence genes of ETEC, EPEC, or STEC. Whole milk was provided ad libitum. After 1‐day acclimation, piglets were randomly assigned to mock‐inoculated (*n* = 3) or ETEC‐challenged (*n* = 3) groups. The ETEC group received 2 mL of LB broth containing PT4357 (5 × 10^8^ CFU/mL) orally, while mock piglets received sterile LB broth. Clinical signs and fecal consistency were assessed every 6 h. All procedures were approved by the Institutional Animal Care and Use Committee (IACUC) of National Taiwan University (NTU‐111‐EL‐00070).

### 2.11. Evaluation of Pathogenicity

After bacterial inoculation, fecal condition was observed every hour. The fecal scoring method was described in our previous study [22], in which the diarrhea condition was divided into four levels: 0, normal stool; 1, loose consistency of the stool; 2, semifluid consistency of the stool; and 3, watery diarrhea. Since all ETEC‐inoculated piglets exhibited diarrhea with score 2–3 at 6 h postinoculation, all piglets in both groups were euthanized by electric shock (110 V and 30 s). Gross lesions were recorded, and intestinal tissues were collected, fixed in 10% neutral‐buffered formalin, paraffin‐embedded, sectioned at 3 μm, and stained with hematoxylin and eosin (H&E), Brown–Hopps (B&H), and Warthin–Starry. Jejunum, ileum, cecum, and colon segments were collected for bacterial isolation. Samples were streaked on tryptic soy agar with 5% sheep blood and incubated at 37°C overnight, and hemolytic colonies were screened for ETEC virulence genes by multiplex PCR.

## 3. Results

### 3.1. Determination of Virulence Factors of the Pathogenic *E. coli* Strain

The pathogenic *E. coli* PT4357 isolate exhibited β‐hemolysis and was shown by conventional multiplex PCR to harbor the virulence genes encoding F4 fimbriae, LT, and STb. WGS of PT4357 was successfully performed using NGS, and the data were deposited in the NCBI Sequence Read Archive (SRA) under Accession Number SRR34901564. Serotyping classified the isolate as O8:H7. MLST assigned PT4357 to sequence type ST2521, with 100% identity in adk_6, fumC_19, gyrB_3, icd_135, mdh_11, purA_8, and recA_6 loci. For the Pasteur scheme, the PT4357 ETEC was similar to ST1107 and ST1110 with 100% identity in dinB_50, icdA_354, polB_10, putP_5, trpA_7, trpB_4, and uidA_5. However, there was a mismatch in pabB_295, leading to 99.7863% identity [23].

### 3.2. Genomic Characterization and AMR Profiling

WGS of strain PT4357 was performed using Oxford Nanopore sequencing. Genome assembly was generated using Autocycler and consisted of seven contigs with a total genome size of 5,517,431 bp. The assembly exhibited an N50 value of 4,992,895 bp, an L50 of 1, and a GC content of 50.4%. Besides the chromosome, the remaining six plasmid genome sizes are 198,964 bp, 153,821 bp, 97,677 bp, 63,423 bp, 7464 bp, and 3187 bp, respectively. Notably, a CS1‐like CU fimbrial operon at chromosome position 2144187–2149294, namely, CS33, was identified.

Resistome analysis revealed multiple AMR genes, including *emrD*, *marR*, *tetA*, *mdtA*, and *mcr-1*, along with chromosomal mutations associated with quinolone resistance (Table 2). The genome also contained IncFIA plasmid replicons and fimbrial genes (*faeG*, *fimH*, and *fimA*), as well as virulence‐associated genes, including *hemolysin A* and *shiA*, a gene linked to immune evasion originally identified in the Shigella SHI‐2 pathogenicity island (Table 3). PathogenFinder analysis predicted PT4357 to be potentially pathogenic to humans, with an average prediction score of 0.9799 (SD = 0.0153) across four neural networks [24].

**Table 2:** The antimicrobial susceptibility testing result of the ETEC PT4357 strain.

Antimicrobial class	Antimicrobial agent	Interpretation	Resistance‐associated gene	Location
Penicillins	Amoxycillin	NWT	*blaTEM-1B*	Plasmid
β‐Lactam/β‐lactamase inhibitor	Amoxicillin–clavulanate	NWT	*—*	—
Cephalosporins	Cefazolin	ID	*blaTEM-1B*	Plasmid
	Cefalexin	NWT	*blaTEM-1B*	Plasmid
	Cefoxitin	WT	*—*	—
Carbapenems	Imipenem	WT	*—*	—
	Meropenem	NWT	*—*	—
Quinolones	Ciprofloxacin	WT	*gyrA S83L*	Chromosome
	Enrofloxacin	NWT	*gyrA S83L*	Chromosome
Aminoglycosides	Amikacin	WT	*—*	—
	Gentamicin	NWT	*aac*(*3*)*-IV*	Plasmid
	Kanamycin	NWT	*aph*(*3′*)*-Ia*	Plasmid
Tetracyclines	Doxycycline	NWT	*tet*(*A*)	Plasmid
	Tetracycline	NWT	*tet*(*A*)	Plasmid
Phenicols	Chloramphenicol	ID	*floR*	Chromosome
	Florfenicol	NWT	*floR*	Chromosome
Folate pathway inhibitors	SXT	NWT	*sul1*, *sul2*, *dfrA17*, *dfrA1*	Plasmid
Polymyxins	Colistin	NWT	*mcr-1.1*	Plasmid

Abbreviations: ID, insufficient data; NWT, nonwild type; WT, wild type.

**Table 3:** The detection of virulence factors and CS33 major subunit sequences in Taiwan porcine *Escherichia coli* strains.

Type of strains	Number (strain)	Origin	Hemolysis	K88 (F4)	K99 (F5)	F6	F18	F41	LT	STa	STb	STx2e	Eae	CS33 major subunit
ETEC	PT4357	Intestine	+	+	−	−	−	−	+	−	+	−	−	+
Hybrid STEC/ETEC	24‐460	Intestine	+	−	−	−	+	−	−	+	+	+	−	+
Hybrid STEC/ETEC	25‐137	Intestine	+	−	−	−	+	−	−	+	+	+	−	−
Hybrid STEC/ETEC	25‐437	Intestine	+	−	−	−	+	−	+	+	+	+	−	+
Hybrid STEC/ETEC	25‐422	Intestine	−	−	−	−	+	−	−	+	−	+	−	−
ETEC	25‐033	Intestine	+	−	−	−	+	−	−	+	+	−	−	−
ETEC	25‐094	Intestine	+	+	+	−	−	−	−	+	+	−	−	+
ETEC	25‐448	Intestine	+	−	−	−	+	−	−	+	+	−	−	−
ETEC	25‐473	Intestine	−	−	−	−	−	−	−	−	+	−	−	−
ETEC	25‐484	Intestine	+	−	−	−	−	+	−	−	−	−	−	−
EPEC	24‐467	Intestine	−	−	−	−	−	−	−	−	−	−	+	+
*E. coli*	24‐447	Lung	−	−	−	−	−	−	−	−	−	−	−	−
*E. coli*	25‐366	Brain	+	−	−	−	−	−	−	−	−	−	−	−
*E. coli*	25‐445	Intestine	−	−	−	−	−	−	−	−	−	−	−	−
Preliminary screening result			9/14	2/14	1/14	0/14	6/14	1/14	2/14	7/14	8/14	4/14	1/14	5/14

### 3.3. Antimicrobial Susceptibility Testing

The ETEC PT4357 strain exhibited a multidrug‐resistant (MDR) phenotype, demonstrating resistance to 12 of the 18 antimicrobial agents tested (Table 2). The isolate exhibited a nonwild‐type (NWT) phenotype to multiple antimicrobial classes, including penicillins, cephalosporins, carbapenems (meropenem), enrofloxacin, aminoglycosides, tetracyclines, phenicols, folate pathway inhibitors, and polymyxins, whereas it remained wild type (WT) to cefoxitin, imipenem, ciprofloxacin, and amikacin. The isolate carries several acquired resistance genes, including *gyrA S83L* and *floR* and several plasmid‐borne genes (e.g., *blaTEM-1B*, *tet*(*A*), *aac*(*3*)*-IV*, *aph*(*3′*)*-Ia*, *sul1*, *sul2*, *dfrA17*, *dfrA1*, and *mcr-1.1*).

### 3.4. Usher Protein Phylogenetic Analysis

Phylogenetic analysis of sequences of usher proteins indicates that our CS33 pili of the ETEC PT4357 strain belongs to the α clade and was closely related to a published *E. coli* SE11 CS1‐like strain (GenBank Accession Number AP009240.1) (Figure 1) but distinguished from those well‐characterized usher proteins derived from CS1, CS17, or CS19 in the same α clade.

### 3.5. Comparative Sequence and Structural Analyses of the Novel CS33 Pili Carrying ETEC PT4357 With the Human ETEC ESEI_164 CS1 Operons

**Figure 2:**
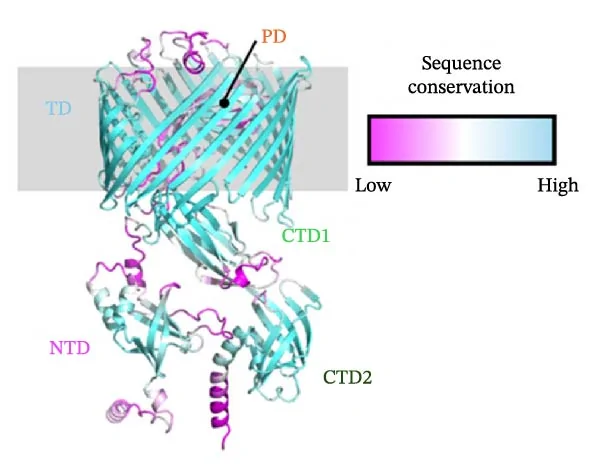
Structural mapping of sequence conservation of CS33. The cartoon representation of apo CS33 was color‐ramped from magenta to white to cyan corresponding to sequence conservation from low to high. The individual domains are indicated with the position of the membrane bilayer shown in a gray rectangle.

Sequence comparison of the operon of the CS33 pili of ETEC PT4357 with the human ETEC ESEI_164 CS1 operon demonstrated a conserved CU fimbrial backbone with marked divergence in surface‐exposed adhesin‐associated components. At the nucleotide level, the pairwise identity across the four core genes ranged from 40.153% to 45.722%. The highest nucleotide conservation was observed in the chaperone gene (45.722%), followed closely by the major subunit (45.560%) and usher gene (44.481%), whereas the minor subunit displayed the lowest identity (40.153%). A similar trend was observed at the amino acid level, where identities were lower overall (29.43%–33.84%). The chaperone protein showed the greatest amino acid conservation (33.84%), while the major subunit, usher, and minor subunit shared 30.18%, 30.54%, and 29.43% identity, respectively. Structural superposition between the AlphaFold3 models of CS33 and human ETEC CS1 further supported this interpretation. The major subunit yielded the lowest RMSD value (0.667 Å), indicating a highly conserved tertiary architecture despite modest sequence identity (Figure 2). Likewise, the chaperone exhibited a low RMSD of 1.160 Å, consistent with preservation of the canonical immunoglobulin‐like donor‐strand complementation machinery required for pilus biogenesis. In contrast, the usher protein showed greater structural divergence (RMSD 1.892 Å). The most divergent component was the minor subunit, which had the highest RMSD (2.453 Å).

**Figure 3:**
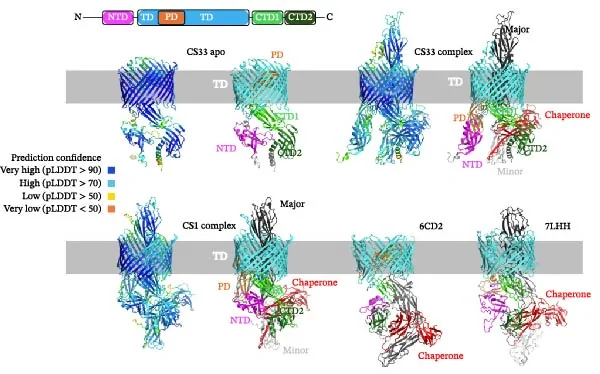
AlphaFold modeling of CS33 and CS1 with and without their binding partners. The domain architectures of CS33 and CS1 are illustrated in the top left corner with matching colors in the cartoon representations of the AlphaFold models and the crystal structure of the *E. coli* PapC usher bound to the chaperone‐adhesin PapD‐PapG (PDB entry 6CD2 https://www.rcsb.org/structure/6CD2) and the cryo‐EM structure of *E. coli* P pilus tip assembly intermediate PapC‐PapD‐PapK‐PapG (PDB entry 7LHH https://www.rcsb.org/structure/7LHH). The N‐terminal periplasmic domain (NTD), the plug domain (PD), and the C‐terminal periplasmic domain (CTD1/2) would undergo significant conformational rearrangements upon binding to the chaperone and substrates, as illustrated in different functional states of CS33 (apo versus complex forms) and the two experimental structures. The individual domains are indicated with matching color as defined above; the position of the membrane bilayer is shown in a gray rectangle. For the AlphaFold models, the prediction confidence scores, predicted local distance difference test (pLDDT), were color‐ramped in reverse rainbow scale with their definitions indicated on the left inset.

Comparing structural models of CS33 and CS1 (Figure 3), both proteins consist of an N‐terminal periplasmic domain (NTD), a membrane‐embedded translocator domain (TD), a passenger domain (PD), and two C‐terminal domains (CTD1 and CTD2). The TD forms a stable β‐barrel within the membrane and shows the highest prediction confidence. In the CS33 apo form, the TD is well‐structured, while extracellular regions remain flexible. Upon complex formation, these domains become more ordered through interactions with major, minor, and chaperone components, indicating stabilization upon assembly. The CS1 complex exhibits a similar architecture but differs in the orientation of its passenger and C‐terminal domains, along with a distinct pattern of chaperone interaction. The periplasmic (pericytoplasmic) domains, mainly involving the NTD and parts of CTD2, show lower confidence and higher flexibility in apo forms of both proteins. Overall, CS33 and CS1 share a conserved core but differ in extracellular and periplasmic domain organization.

### 3.6. Preliminary Screening of CS33 Major Protein Gene in Porcine *E. coli* Strains

Among 14 Taiwan porcine *E. coli* field isolates, the CS33 major protein gene was detected in five *E. coli*, including two in hybrid STEC/ETEC, two in ETEC, and another one in EPEC. Interestingly, no CS33 pilus major subunit gene was detected in the two *E. coli* strains devoid of these virulence genes (Table 1).

### 3.7. Bacterial Adhesion Assay

**Figure 4:**
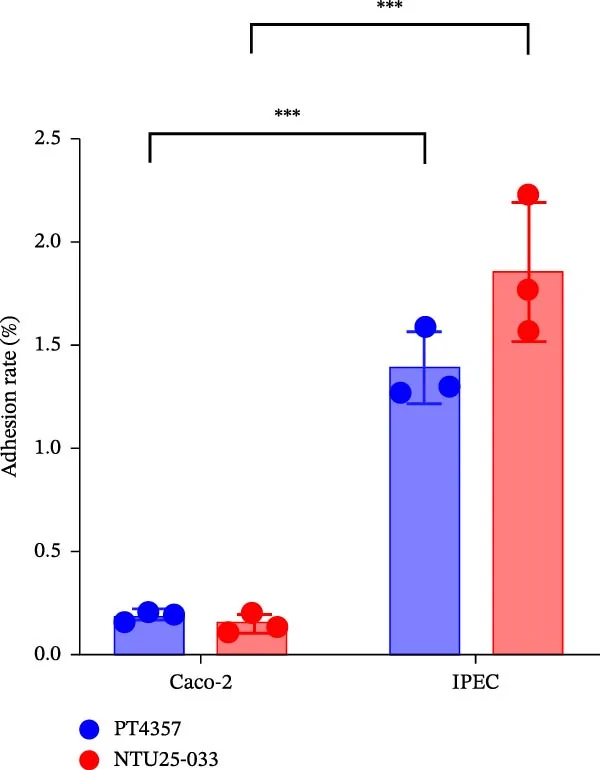
The adherence of PT4357 (CS33 pili–expressing) and NTU25‐033 (CS33‐negative) *Escherichia coli* strains in porcine IPEC and human Caco‐2 cells. All assays were performed in triplicate, and statistical analysis was conducted using Prism software.  ^∗∗∗^ *p* < 0.001 indicates a highly significant difference was observed.

The porcine ETEC strains PT4357 (CS33‐positive) and NTU25‐033 (CS33‐negative) were selected for the in vitro adhesion assay. In human Caco‐2 cells, these two ETEC strains exhibited about 0.2% adherence rates, showing no significant difference. In contrast, in porcine IPEC cells, both ETEC strains exhibited around 1.3%−1.7% (Figure 4) adherence rates that were significantly higher than those in human Caco‐2.

### 3.8. Pathogenicity of the ETEC PT4357 Strain in Neonatal Piglets

The virulence of ETEC PT4357 was assessed in 4‐day‐old neonatal piglets. All mock‐inoculated pigs maintained normal fecal consistency and clinical health throughout the study, whereas all ETEC‐challenged piglets developed lethargy, inappetence, vomiting, and severe diarrhea (scores 2–3) within 6 h postinoculation (Table 4). Gross examination revealed dilation of the small intestine and cecum with watery intestinal contents in challenged piglets, whereas the mock group exhibited normal intestinal morphology without notable lesions.

**Table 4:** Fecal consistency scoring of piglets in the ETEC PT4357–inoculated group and mock‐inoculated group.

Piglet number		ETEC PT4357–inoculated group			Mock‐inoculated group		
		1.	2.	3.	4.	5.	6.
Hours postinoculation	−16	0	0	0	0	0	0
	−10	0	0	0	0	0	0
	−4	0	0	0	0	0	0
	2	0	0	0	0	0	0
	6	2	3	3	0	0	0

*Note:* Three piglets at 4 days old were orally inoculated with 2 mL of LB broth containing the ETEC PT4357 strain at a final concentration of 5 × 10⁸ CFU/mL, while piglets in the mock group received 2 mL of sterile LB broth. Scores of 0, 1, 2, and 3 indicate normal, muddy, semiliquid, and liquid, respectively.

**Figure 5:**
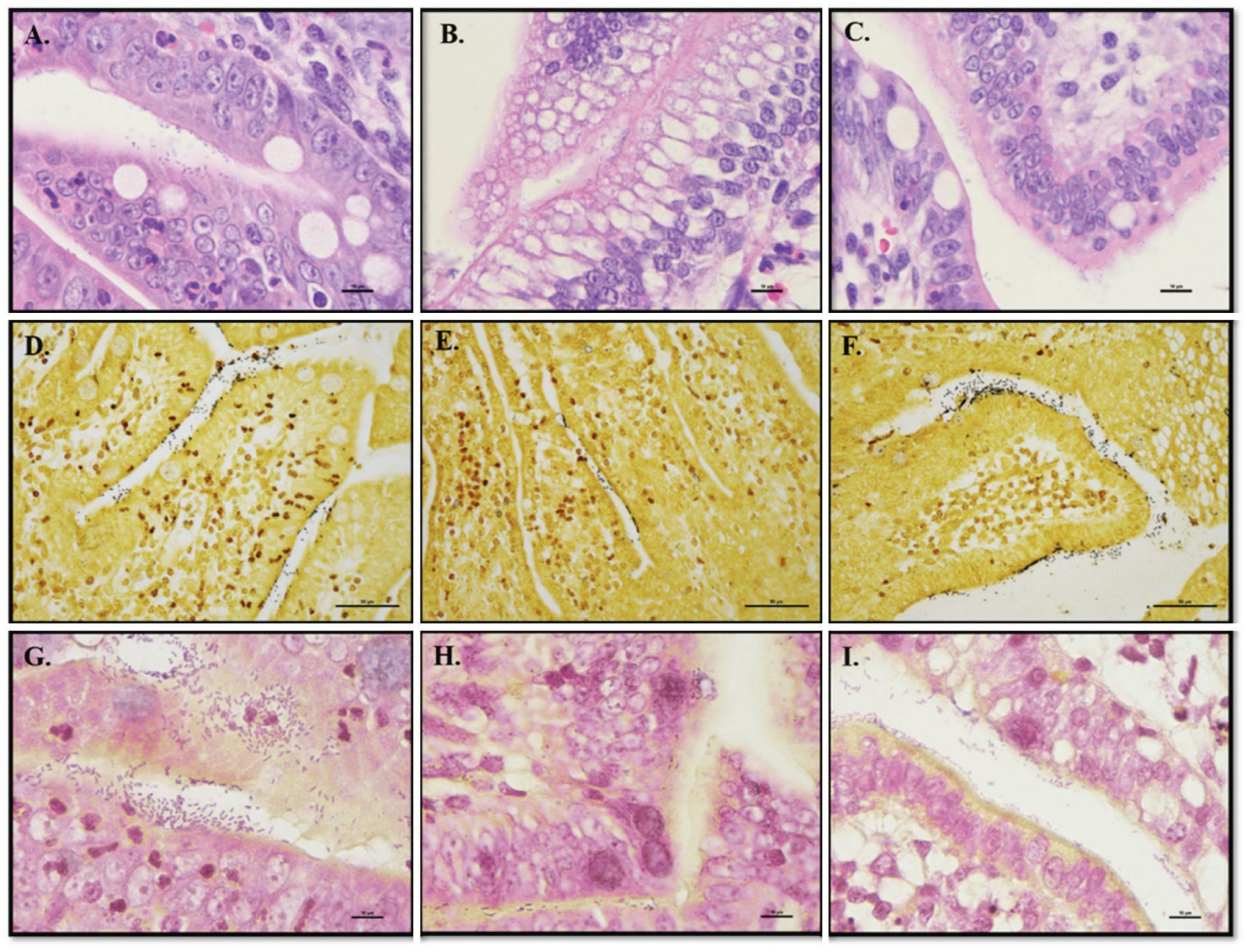
Histopathological evaluation of the sections from duodenum, jejunum, and ileum in the ETEC PT4357–inoculated group by H&E stain (A–C), Warthin–Starry stain (D–F), and Brown–Hopps (B&H) stain (G–I). (A) Duodenum (1000x; bar = 10 µm). (B) Jejunum (1000x; bar = 10 µm). (C) Ileum (40x; bar = 500 µm). (D) Duodenum (40x; bar = 500 µm). (E) Jejunum (40x; bar = 500 µm). (F) Ileum (40x; bar = 500 µm). (G) Duodenum (1000x; bar = 10 µm). (H) Jejunum (1000x; bar = 10 µm). (I) Ileum (1000x; bar = 10 µm).

Histopathological evaluation revealed no significant microscopic lesions in either group, except for dense colonies of bacilli adhering to the surface of small intestinal villi in challenged piglets (duodenum, jejunum, and ileum; Figure 5). B&H and Warthin–Starry staining confirmed these clusters as gram‐negative bacteria attached to enterocytes. No bacterial adherence was observed in the cecum and colon or in any sections of mock‐inoculated piglets.

### 3.9. Bacterial Reisolation

Bacterial isolation from intestinal segments of challenged piglets confirmed the presence of β‐hemolytic colonies in the jejunum and ileum. Multiplex PCR analysis verified that the reisolated colonies carried the same virulence genes of F4, LT, and STb toxins as the original PT4357 inoculum, confirming successful colonization and reproducibility of the infection model.

## 4. Discussion and Conclusion

F4 ETEC is a major cause of neonatal diarrhea in piglets, but experimental studies on intestinal tropism remain limited. In this study, a neonatal piglet oral challenge model was used to examine the pathogenicity, colonization, and underlying mechanisms of the field‐isolated F4 ETEC PT4357 strain. Consistent with previous reports [4, 25], the F4 ETEC PT4357 induced severe watery diarrhea within 6 h, confirming its virulence. Colonization was observed on villous surfaces throughout the small intestine but was absent in the cecum and large intestine, suggesting that the small intestine is the primary site of adherence. Histopathology revealed bacilli adhering to enterocytes without overt microscopic lesions or inflammatory responses might reflect the acute nature of infection in the field.

In the present study, WGS of ETEC PT4357 identified multiple adhesion‐associated genes. The plasmid‐encoded F4 (K88) fimbrial operon (*faeB*) is the primary colonization factor, mediating receptor‐specific attachment to porcine enterocytes. Additional adhesion‐associated genes, including type 1 fimbriae (*fimA* and *fimH*), Yeh fimbriae (*yehABCD*), F19 fimbriae (*papA_F19*), long polar fimbriae (*lpfA*), *tia*, *fdeC*, and *yghJ*, are predicted to enhance bacterial attachment, mucus penetration, and intestinal colonization [26, 27]. The strain also carries three plasmid‐encoded enterotoxins, LTIh‐4 (*eltIAB-4*), STb1 (*estb*), and EAST‐1 (*astA*), which are associated with secretory diarrhea through the disruption of intestinal fluid and electrolyte homeostasis [28, 29]. Additional virulence‐associated genes further support pathogenicity. The biofilm‐associated gene *csgA* may promote environmental persistence, while *iss* and *traT* might enhance resistance to complement‐mediated killing. The hemolysin genes *hlyA* and *hlyE* may contribute to epithelial injury and facilitate bacterial dissemination. Stress adaptation genes, including *gad*, *shiA*, and *anr*, are likely to improve survival under gastrointestinal conditions, whereas *terC* may increase the resistance to oxidative stress. Furthermore, the plasmid‐encoded conjugation regulator *traJ* suggests the potential for horizontal dissemination of virulence determinants [27, 30]. Collectively, these genomic features predict that ETEC PT4357 possesses a high pathogenic potential and is well adapted for efficient colonization and induction of severe diarrheal disease in neonatal piglets.

Interestingly, a CS1‐like CU fimbrial operon, namely, CS33, is the first report of this locus in porcine ETEC. Alignment and phylogenetic analysis of usher protein sequences demonstrated that the novel CS33 CU pilus of the porcine ETEC strain PT4357 clusters within the α clade and is most closely related to the *E. coli* SE11 CS1‐like usher (AP009240.1) yet remains clearly distinct from the canonical CS1, CS17, and CS19 usher proteins. This positioning suggests that CS33 represents a divergent lineage within a conserved CU fimbrial framework. Comparative genomic and structural analyses further revealed that the CS33 operon retains the conserved CU assembly backbone but exhibits substantial divergence in surface‐exposed and adhesin‐associated components, with nucleotide identities ranging from ~40% to 46% and amino acid identities reduced to ~29%–34% relative to the human ETEC CS1 operon. Despite this sequence variability, structural modeling showed strong conservation of the major subunit and chaperone folds. In contrast, the usher and, in particular, the minor subunit displayed greater structural divergence. Based on sequence diversity and structural modeling, we propose the nomenclature CS33C, CS33B, CS33A, and CS33D for the usher, chaperone, major, and minor subunits of the α‐clade CS33 CU assembly pilus, respectively. Overall, the CS33 CU pilus appears to maintain a conserved biogenesis architecture while evolving distinct extracellular features.

While CS1‐like pili have been detected in diverse pathogenic and commensal *E. coli* [31], our preliminary screening results revealed that the CS33 major subunit gene was detected in 35.7% Taiwan porcine *E. coli* strains, including porcine ETEC, hybrid STEC/ETEC, and EPEC isolates but not in the lung and brain isolates without carrying enteric virulent genes. The results suggest a potential role of this chromosome‐encoded CS33 CU pathway pilus in host specificity and intestinal colonization in pigs. To investigate the role of the CS33 CU pathway pilus–bearing ETEC in both pigs and humans, in vitro adhesion assays using human Caco‐2 cells and porcine IPEC cells, as well as in vivo piglet infection experiments, were performed. Our results showed that the CS33‐positive strain PT4357 exhibited markedly greater adhesion to porcine IPEC cells (1.3%–1.7%) than to human Caco‐2 cells (~0.2%), indicating a preference for the porcine intestinal epithelium and minimal tropism toward the human intestinal epithelium. Furthermore, because both CS33‐positive and CS33‐negative ETEC strains displayed comparable levels of adhesion to porcine IPEC cells, our current data do not support that CS33 itself mediates bacterial attachment to porcine intestinal epithelial cells. Additional studies, including construction of a CS33 knockout mutant, complementation assays, and expression analyses, will be required to determine the specific role of CS33 in the adhesion and colonization of porcine ETEC. This in vitro preference was further corroborated by in vivo pathogenicity, as neonatal piglets developed rapid‐onset severe diarrhea, systemic clinical signs, and characteristic intestinal colonization within hours of challenge. Dense bacterial adherence to small intestinal villi, confirmed by histological staining, together with the successful reisolation of F4^+^, LT^+^, and STb^+^ strains, confirms efficient intestinal colonization and virulence expression in the natural host [32–34]. The results strongly indicate that the CS33‐positive ETEC PT4357 is a highly pathogenic porcine strain but exhibits minimal adhesion to and colonization of human intestinal epithelial cells in vitro. However, its potential pathogenicity in humans requires further investigation.

MLST classified PT4357 as ST2521, previously reported in Taiwan [35] and the United States [36], and strongly associated with the plasmid‐mediated colistin resistance gene *mcr-1*, observed in this strain. PT4357 carried an IncFIA plasmid, which in other Enterobacteriaceae can harbor *blaNDM-1*; however, WGS confirmed its absence, consistent with susceptibility to meropenem and imipenem. Additional resistance genes, including *tet*(*A*), *blaTEM-1B*, *sul2*, and *dfrA1*, were concordant with the observed phenotypic resistance profiles, supporting the genomic findings and indicating the presence of MDR *E. coli* in swine. Most acquired resistance determinants, including *blaTEM-1B*, *tet*(*A*), *aac*(*3*)*-IV*, *aph*(*3′*)*-Ia*, *sul1*, *sul2*, *dfrA17*, *dfrA1*, and *mcr-1.1*, were plasmid‐borne, suggesting a high potential for horizontal transfer and dissemination of AMR, while the presence of the transferable colistin resistance gene *mcr-1.1* is of particular public health concern. A limitation of this study is that quinolone resistance was assessed solely by phenotypic antimicrobial susceptibility testing using MICs and disk diffusion interpreted according to the ECOFFs. Overall, the AMR genotype was largely consistent with the phenotypic susceptibility profile, particularly for *mcr-1.1*, which correlated with the NWT phenotype to colistin, demonstrating good genotype–phenotype concordance. However, minor discrepancies were observed for *blaTEM-1B* and *floR*. Although *blaTEM-1B* was detected, cefazolin was classified as insufficient data (ID) and cephalexin as NWT. This may reflect the substrate specificity of TEM‐1, which primarily hydrolyzes penicillins and early‐generation cephalosporins, while the degree of phenotypic resistance is influenced by β‐lactamase expression levels, inoculum effects, and outer membrane permeability [37, 38]. Likewise, although *floR*, which encodes an efflux pump conferring resistance to both chloramphenicol and florfenicol, was identified, chloramphenicol was classified as ID, whereas florfenicol was NWT. This difference may be attributable to variable *floR* expression, differences in substrate affinity, or the limited availability of validated ECOFFs [39]. In the present study, ciprofloxacin was WT, whereas enrofloxacin was NWT despite the detection of the quinolone resistance‐associated mutation *gyrA S83L* in ETEC PT4357. Quinolone resistance in *E. coli* is primarily driven by accumulation of chromosomal mutations in the quinolone resistance‐determining regions (QRDRs) of *gyrA*, *gyrB*, *parC*, or *parE*; plasmid‐mediated quinolone resistance determinants (e.g., *qnr*, *aac*(*6′*)*-Ib-cr*, *qepA*, and *oqxAB*); or alterations in efflux pump activity and outer membrane permeability [40]. A single substitution at codon 83 of GyrA typically results in only a modest increase in the MIC, and its phenotypic impact may differ among fluoroquinolones because of differences in their intrinsic activity and the corresponding ECOFFs used for interpretation. Consequently, the MIC may exceed the VetCAST ECOFF for enrofloxacin while remaining within the WT distribution for ciprofloxacin.

In the present study, although all three infected piglets showed symptoms and similar tissue tropism, limitations remain: The sample size was small, SPF pigs were used, and the high inoculation dose may have accelerated disease onset. These conditions may not reflect field infections, requiring further validation studies under natural farm settings more broadly. Genomic characterization revealed key virulence factors and multiple AMR determinants, highlighting public health concerns. The neonatal piglet model established here provides a reliable platform for studying host‐pathogen interactions and evaluating preventive or therapeutic strategies against ETEC infections, emphasizing the importance of monitoring AMR in swine populations. Our results indicate that porcine *E. coli* may harbor the mcr‐1 antibiotic resistance gene and also possess diverse CU pathway pili, highlighting the need for further molecular investigation and epidemiological surveillance.

**Nomenclature**
ETEC: Enterotoxigenic *Escherichia coli*
*E. coli*: *Escherichia coli*
PWD: Postweaning diarrhea
ED: Edema disease
LT: Heat‐labile toxin
ST: Heat‐stable toxin
cAMP: Cyclic adenosine monophosphate
cGMP: Cyclic guanosine monophosphate
NGS: Next‐generation sequencing
SRA: Sequence Read Archive
MLST: Multilocus sequence typing
AMR: Antimicrobial resistance
MDR: Multidrug‐resistant
B&H: Brown and Hopps
LT‐I: Heat‐labile enterotoxin I
SXT: Trimethoprim‐sulfamethoxazole
EPEC: Enteropathogenic *Escherichia coli*
STEC: Shiga toxin–producing *Escherichia coli*
H&E: Hematoxylin and eosin
CLSI: Clinical and Laboratory Standards Institute
PBS: Phosphate‐buffered saline
CDS: Coding sequences
EC: Enzyme Commission
COG: Clusters of Orthologous Groups
VFDB: Virulence Factor Database
CGE: Center for Genomic Epidemiology
CARD: Comprehensive Antibiotic Resistance Database
IACUC: Institutional Animal Care and Use Committee
CU: Chaperone‐usher.

## Author Contributions

Formal analysis, investigation, methodology, writing – original draft preparation: Yi‐Fang Lai and Wei‐Ting Lee. Data curation, resources, supervision: Shang‐Te Danny Hsu, Yen‐Chen Chang, Chao‐Nan Lin, Tzu‐Ming Huang, Dung‐Chi Wu, Hao‐Sen Chiang, Chien‐Yu Chen. Conceptualization, formal analysis, supervision, project administration, validation, visualization, writing – review and editing: Hui‐Wen Chang.

## Funding

This work was supported by the National Science and Technology Council, Taiwan, R.O.C. (Grant NSTC 115‐2321‐B‐002‐027 for Hui‐Wen Chang). Shang‐Te Danny Hsu is supported by the Academia Sinica intramural fund, an Academia Sinica Investigator Award (Grant AS‐IV‐114‐L04), and the National Science and Technology Council (NSTC), Taiwan (Grants 114‐2123‐M‐001‐008‐MY3 and 113‐2311‐B‐001‐01‐MY3).

## Disclosure

All the authors have read and approved the final manuscript.

## Ethics Statement

For the clinical specimen collection, informed consent has been obtained at the time of sample submission from farmers participating in the study. The procedures involving animal experiments were approved and permitted by the Institutional Animal Care and Use Committee (IACUC) of National Taiwan University (NTU; Taiwan, Republic of China) and carried out under the regulations of the IACUC protocol at NTU with the Approval Number of NTU‐111‐EL‐00070. All euthanasia procedures were performed in accordance with the AVMA Guidelines for the Euthanasia of Animals: 2020 Edition (American Veterinary Medical Association, Schaumburg, IL, USA).

## Conflicts of Interest

The authors declare no conflicts of interest.

## Data Availability

The datasets used and/or analyzed during the current study are available from the corresponding author upon reasonable request.
